# Comparison of Reliable Reference Genes Following Different Hormone Treatments by Various Algorithms for qRT-PCR Analysis of *Metasequoia*

**DOI:** 10.3390/ijms20010034

**Published:** 2018-12-21

**Authors:** Jing-Jing Wang, Shuo Han, Weilun Yin, Xinli Xia, Chao Liu

**Affiliations:** 1 National Engineering Laboratory for Tree Breeding, Beijing Forestry University, Beijing 100083, China; wangjingjing@bjfu.edu.cn (J.-J.W.); hanhan@bjfu.edu.cn (S.H.); yinwl@bjfu.edu.cn (W.Y.); xiaxl@bjfu.edu.cn (X.X.); 2 College of Biological Sciences and Technology, Beijing Forestry University, Beijing 100083, China; 3 Key Laboratory of Genetics and Breeding in Forest Trees and Ornamental Plants, Beijing Forestry University, Beijing 100083, China

**Keywords:** reference genes, normalization, hormone treatment, reverse transcription-quantitative PCR, *Metasequoia*

## Abstract

Quantitative reverse transcription polymerase chain reaction (qRT-PCR) is the most sensitive technique for evaluating gene expression levels. Choosing appropriate reference genes for normalizing target gene expression is important for verifying expression changes. *Metasequoia* is a high-quality and economically important wood species. However, few systematic studies have examined reference genes in *Metasequoia*. Here, the expression stability of 14 candidate reference genes in different tissues and following different hormone treatments were analyzed using six algorithms. Candidate reference genes were used to normalize the expression pattern of FLOWERING LOCUS T and pyrabactin resistance-like 8. Analysis using the GrayNorm algorithm showed that *ACT2* (Actin 2), *HIS* (histone superfamily protein H3) and *TATA* (TATA binding protein) were stably expressed in different tissues. *ACT2*, *EF1α* (elongation factor-1 alpha) and *HIS* were optimal for leaves treated with the flowering induction hormone solution, while *Cpn60β* (60-kDa chaperonin β-subunit), *GAPDH* (glyceraldehyde-3-phosphate dehydrogenase) and *HIS* were the best reference genes for treated buds. *EF1α, HIS* and *TATA* were useful reference genes for accurate normalization in abscisic acid-response signaling. Our results emphasize the importance of validating reference genes for qRT-PCR analysis in *Metasequoia*. To avoid errors, suitable reference genes should be used for different tissues and hormone treatments to increase normalization accuracy. Our study provides a foundation for reference gene normalization when analyzing gene expression in *Metasequoia*.

## 1. Introduction

Quantitative reverse transcription polymerase chain reaction (qRT-PCR) is an efficient, sensitive and reliable technique for quantifying the expression profiles of target genes in different tissues, following different hormone treatments and under various stresses [1]. qRT-PCR enables comparison of changes in the expression of a target gene to changes in reference genes, including abundant transcripts and low-abundance transcripts of the target gene. Thus, the qRT-PCR is widely preferred over classic transcriptome analysis methods [2]. Appropriate normalization is a key factor in quantifying transcript expression levels to avoid bias [3]. Normalization requires the selection of one or more reference genes showing constant and stable expression levels in different tissues and following different treatments [4,5]. To eliminate differences in the initial volume of cDNA template, quality of RNA from different tissues and reaction efficiency of enzymes, reference genes are typically required for data correction in qPCR analysis [6].

An ideal reference gene has the following characteristics: first, it is expressed stably in different tissues and cells; second, its expression is not greatly affected by environmental, biological or abiotic stress or other factors; finally, its expression level is similar to that of the target gene. Numerous studies of reference genes have been conducted in model plants and other species, such as *Arabidopsis* [7], *Populus euphratica* [8,9], *Lycopersicon esculentum* [10], *Solanum tuberosum* [11], *Eucalyptus robusta* [12] and Slash Pine [13]. According to previous studies, the most stable reference genes in many species are related to basic cellular processes, such as the genes for actin [14], tubulin, elongation factor 1-α [15], glyceraldehyde-3-phosphate dehydrogenase [16] and 18S ribosomal RNA [17]. However, many studies reported that the expression levels of these genes are not constant under all experimental conditions or in different species. No universal reference gene has been identified [18,19]. Comparative analysis of reference genes showed that widely used reference genes are not stable under different experimental conditions [20,21]. Therefore, to identify an accurate and efficient target gene expression profile for gene expression analysis, we determined the best reference genes in *Metasequoia* for studying different plant tissues and following different hormone treatments before normalization of gene expression.

Several bioinformatics tools including geNorm [22], NormFinder [23] and BestKeeper [24] have been utilized to analyze and assess the expression stability of reference genes for qRT-PCR data normalization. geNorm is a popular algorithm which reveals the most stable reference genes from among many tested candidate reference genes. The NormFinder algorithm identifies the optimal normalization gene among a set of candidates. BestKeeper is an Excel-based tool for selecting the best candidate using pairwise correlations. In addition to these methods, the ΔCt [25] and GrayNorm [26] algorithms have been widely employed for data analysis. The RankAggreg algorithm was used in a comprehensive sequencing study on genetic stability [27].

*Metasequoia glyptostroboides* Hu & Cheng, regarded as the national first-class protected plant, is a “living fossil” discovered as a relict population in a remote area near the border of Hubei in South-Central China in the 1940s [28]. The discovery of *Metasequoia* was one of the most important events contributing to species protection of China in the past century [29]. *Metasequoia* Miki ex Hu et Cheng belongs to the Cupressaceae family and *Metasequoia glyptostroboides* Hu & Cheng is the only species in this genus. *Metasequoia* is a coniferous tree species widely distributed in southern China and is considered as an ideal species for forest evolution studies. It is a high-quality timber species because it has conserved various characteristics of ancient trees but also evolved unique features over its long evolution [30,31]. The unique wood features of *Metasequoia* including its beautiful and delicate texture, durability and weightlessness [32]. It is widely used as a raw material for producing floors, walls and furniture. In addition to these applications, *Metasequoia* is a popular research material in the fields of pharmacology and biochemistry [33,34]. Although *Metasequoia* has high economic and scientific value, its use is limited by long growth and reproduction times, as observed for other gymnosperms. *Metasequoia* requires approximately 25 years to form the first female core. After a long juvenile period, the core matures and can form flower buds. Approximately 45 years are required to form a large amount of fruit [35]. Studies of the molecular mechanisms of floral bud formation and abscisic acid (ABA)-response signaling in this tree species have revealed that ABA promotes the growth and development of this gymnosperm.

Gene expression analyses are important for studying floral bud differentiation and abiotic stress in gymnosperms [36]. The conversion from vegetative growth to reproductive growth is an important process in plant development and key event in plant reproduction. This process is regulated by both external environment and internal factors. In this study, we sought to evaluate candidate reference genes in *Metasequoia*, a valuable resource for examining gene expression levels, for the first time. Our results provide a foundation for other researchers to choose reference genes for the normalization of mRNA levels by qRT-PCR in this tree species.

## 2. Results

### 2.1. Primer Specificity and Expression Analysis of 14 Candidate Reference Genes and Two Target Genes

**Figure 1:**
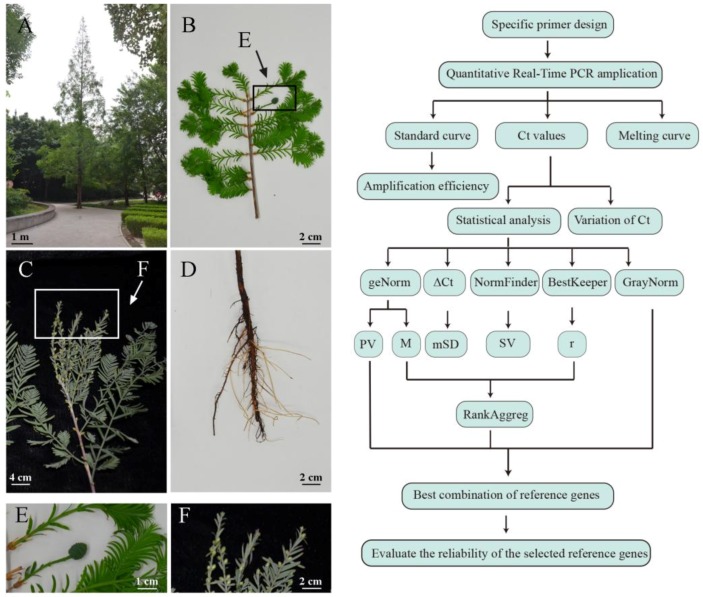
Sample sets and data analysis flow chart. *Metasequoia* tree (**A**), branch with female cones (**B**), branch with male cones (**C**), root (**D**), female core (**E**), male core (**F**), flow chart (**G**). Abbreviations: SV: stability values; mSD: mean standard deviation; r: correlation coefficient; PV: pairwise variation; M: M-values.

To investigate the mechanism of hormone-induced gymnosperm flowering and the pathway of ABA, we evaluated selected candidate reference genes in the present study. We validated 14 potential reference genes in different tissues such as the leaf, stem, root, bud, male core and female core. The potential reference genes were actin 2 (*ACT2*), AP-2 complex subunit mu-like (*AP-2*), 60-kDa chaperonin β-subunit (*Cpn60β*), elongation factor-1α (*EF1*α), eukaryotic initiation factor 5A (*elF-5A*), glyceraldehyde-3-phosphate dehydrogenase (*GAPDH*), glucosidase II α-subunit (*GII*α), histone superfamily protein H3 (*HIS*), rubisco activase (*RA*), ribosomal protein L27e (*RP*), ribosomal protein L17 (*RPL17*), TATA binding protein 2 (*TATA*), tubulin β chain (*TUB*) and ubiquitin family 6 (*UBQ*). These genes were used to normalize the expression patterns of flowering locus T (*FT*) and pyrabactin resistance-like 8 (*PYL*). The sample set and data analysis flow chart are shown in Figure 1. 

Only one band of the expected size was detected by 2% agarose gel electrophoresis (Table 1, Figure S1). The specificity of PCR detection was confirmed as a single peak in melting-curve analysis (Figure S2). Therefore, these primer pairs were used in the next qRT-PCR experiments. The PCR efficiency varied from 1.8518 for *RP* to 2.1419 for *AP-2* (Table 1).

**Figure 2:**
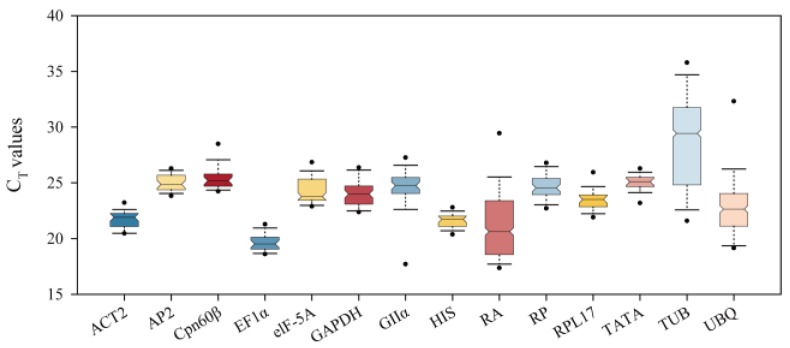
Expression stability of the tested 14 reference genes in all 19 samples. The line shown in the box indicates the median Ct value. The upper edge of each box indicates the 25th percentile and lower indicates the 75th percentile.

The Ct values are listed in Supplementary Table S1 and reveal the different gene expression levels. The minimum Ct value of the 14 reference genes was 17.30 for *RA*, while the maximum value was 35.87 for *TUB*. The Ct value of each candidate reference gene is presented in Figure 2. In different *Metasequoia* tissues and organs, the Ct values varied from 18.80 to 35.87, following flowering induction hormone treatments, the Ct values ranged from 18.98 to 31.99 and the Ct values ranged from 17.36 to 34.77 following ABA treatments.

**Figure 3:**
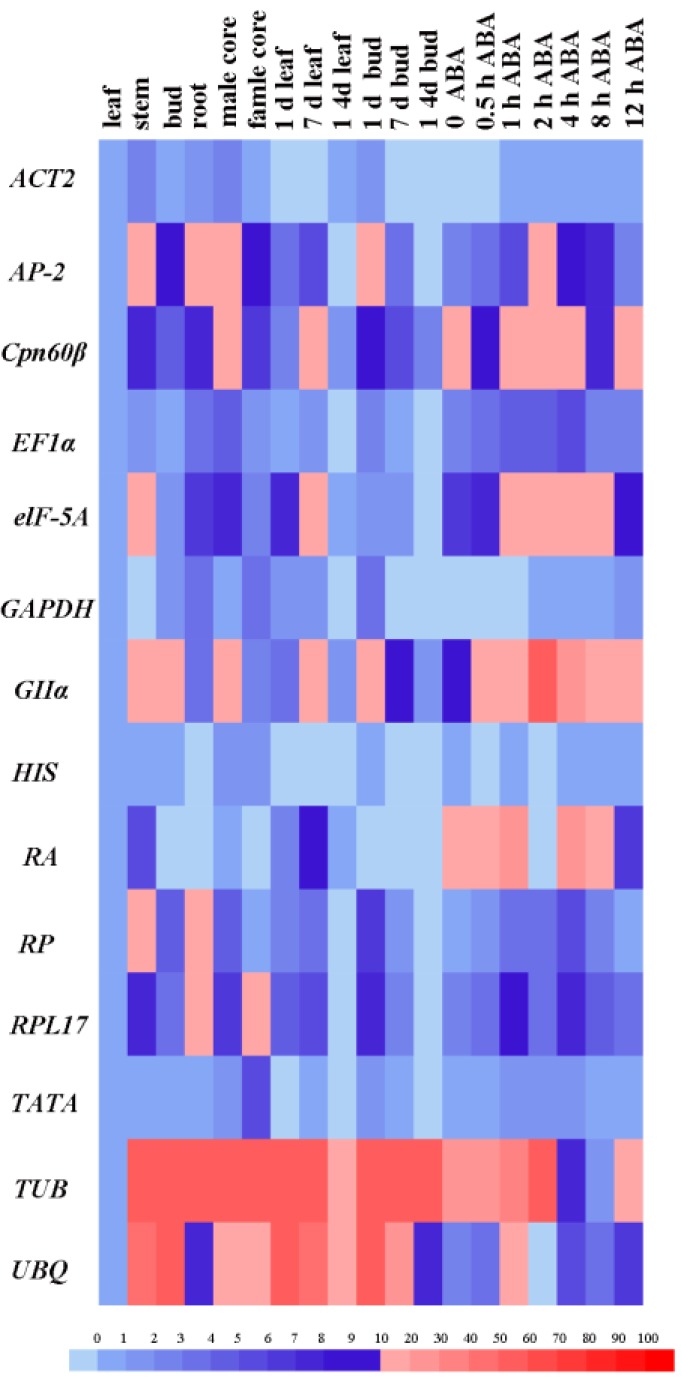
Heat map showing gene expression levels in all 19 samples. The color scale represents the gene expression level. Low to high expression is represented over a spectrum from blue to red, respectively. Abbreviations: ABA: Abscisic Acid treatment.

The expression of 14 genes in all 19 samples was calculated using the 2^−ΔCT^ method to analyze expression stability; the expression of each gene in the leaves was measured as a control. The heatmap directly reveals the stability of gene expression in all samples (Figure 3). The expression levels of *ACT2*, *GAPDH*, *HIS* and *TATA* were stable, while *TUB* and *UBQ* showed large variations in expression levels. 

### 2.2. Analysis of Gene Expression Stability

**Figure 4:**
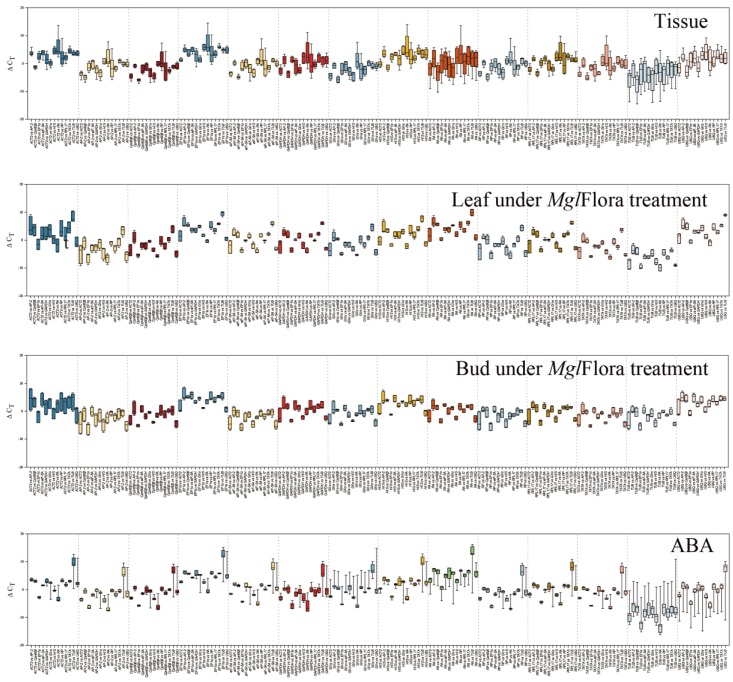
ΔCt method of reference genes in different samples. The Ct variation of the samples between different reference genes is shown in a box-whisker plots. The shorter box indicates that both genes express stably relatively, while, the longer the box indicates that more variable expression of both genes. Abbreviations: ABA: Abscisic Acid treatment.

The mean standard deviation (mSD) can be used to assess whether the expression of a gene is stable. A low mSD value of a candidate reference gene indicates that the gene is expressed stably in different samples. We used this ΔCt approach to screen candidate reference genes (Table 2). The ranking results showed in a box plot visually revealed whether the distribution of ΔCt values between a gene and the other 13 genes was discrete or concentrated. A longer box indicates greater deviation in the ΔCt value, suggesting that one or both genes is changing in the sample and that expression is relatively unstable. In contrast, a shorter box indicates smaller deviations, suggesting relatively stable expression of two genes (Figure 4). 

In BestKeeper analysis, the correlation coefficient (r), standard deviation (SD) and coefficient of variation (CV) indicate the variation among Ct values, which can determine candidate gene stability. In this study, the most stable gene in different tissues was *AP-2*, while the least stable gene was *RA* (Table 3). *HIS* was selected as the most stable gene in leaves following *Mgl*Flora treatment and *Cpn60β* was the most stable in the treated buds, while the least stable gene was *AP-2* (Table 3). In contrast to the ranking order identified by geNorm, *TATA* was the best choice for samples treated with ABA, while *TUB* is the most unstable gene. 

**Figure 5:**
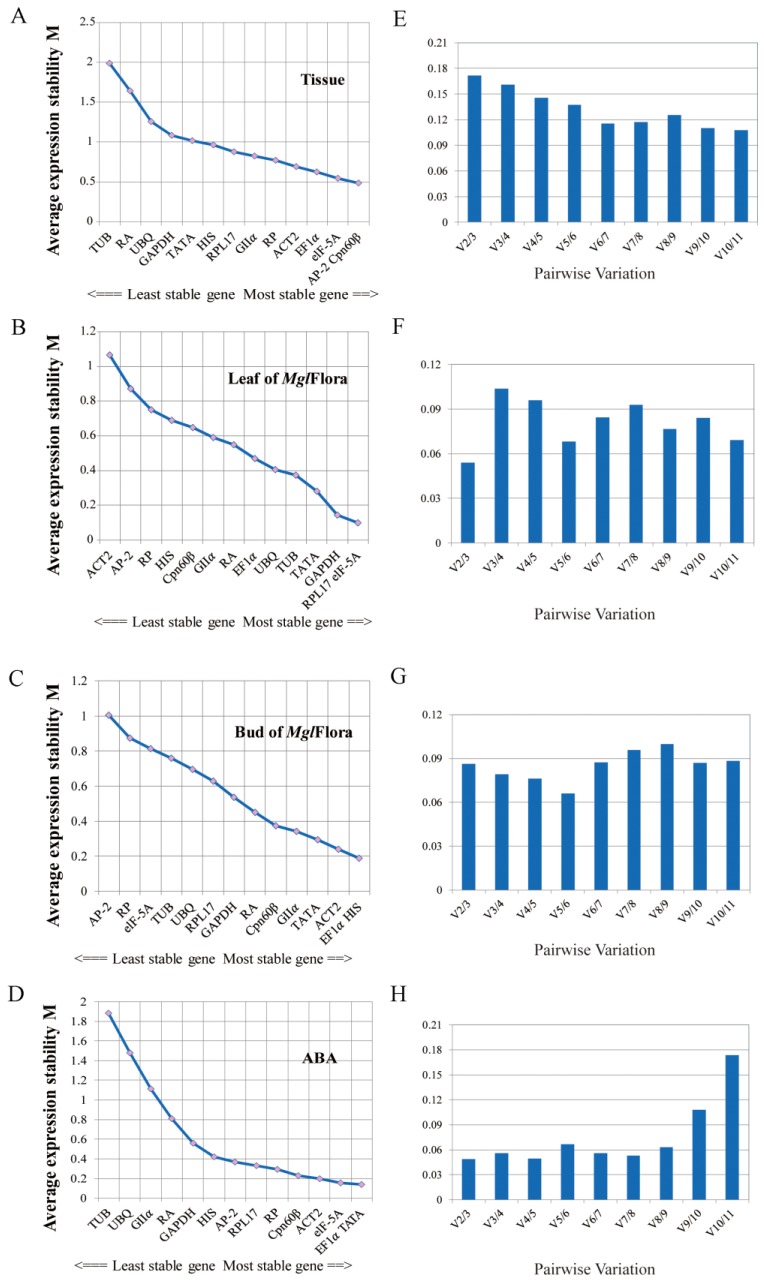
Average gene expression stability (M) and pairwise variation (V) calculated by geNorm. Gene expression stability is an index of the selection of candidate reference genes. Pairwise variation reflects the minimum number of reference genes for normalization by qRT-PCR. Ranking of the expression stability of the 14 genes and pairwise variation in different tissues (**A**,**E**), leaves following *Mgl*Flora treatment (**B**,**F**), buds following *Mgl*Flora treatment (**C**,**G**) and ABA treatment (**D**,**H**). Abbreviations: ABA: Abscisic Acid treatment; M: M-values.

geNorm uses the mean expression stability values (M-values) to determine the reference gene expression stability, which is represented as the average pairwise variation (PV, V) of a specific gene with all other tested candidate genes. Lower M-values indicate greater expression stably, while higher M- values indicate lower expression stability. In our analysis, the most stable genes were *AP-2* and *Cpn60β* with an M-value of 0.478 in different tissues (Table 4; Figure 5A). *RPL17* and *elF-5A* were the most suitable candidate genes for normalization with an M-value of 0.105 in the leaves following *MgFlora* treatment (Table 4; Figure 5B), whereas *EF1α* and *HIS* were the most stable genes with an M-value of 0.19 in treated buds (Table 4; Figure 5C). For ABA treatment, *EF1α* and *TATA* showed stable expression with an M-value of 0.139 (Table 4; Figure 5D).

The geNorm algorithm revealed the appropriate number of reference genes needed for standardization to calculate the pairwise variation between the normalization factors NF_n_ and NF_n+1_ of two sequences (V_n/n+1_). When V_n/n+1_ is less than 0.15, n is the number of genes used in normalization. In this study, all V_2/3_ values were less than 0.15 in the four sample sets and thus the best number for normalization was two (Figure 5E–H). Considering the accuracy of the experiment and previous reports, three reference genes were used in this study for target gene expression normalization in *Metasequoia.*

According to the NormFinder algorithm, the most stable candidate reference genes have low stability values (SV). When the stability value is close to zero, the gene displays stable expression. The NormFinder rankings of the 14 candidate genes are shown in Table 5. In the six different tissues, *AP-2* ranked first with an SV of 0.197, while *TUB* ranked last with an SV of 3.839. *TATA* was found to be the most stable choice for the leaf and bud of with SVs of 0.152 and 0.233, whereas *ACT2* and *AP-2* were the most unstable genes in flowering induction hormone treatments, respectively. *TATA* was the most stable gene in response to ABA treatment with an SV was 0.07, which contrasted the results calculated by the NormFinder algorithm, showing that the gene with the lowest ranking was *TUB* with an SV of 4.255. 

**Figure 6:**
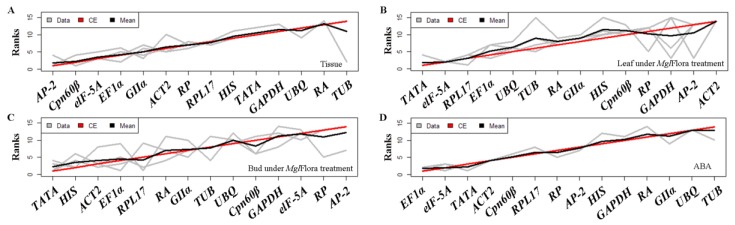
Rank aggregation of 14 candidate reference genes in four sample sets. The RankAggreg package is loaded in the R program. Ranking lists of ΔCt, BestKeeper, NormFinder and geNorm are shown as gray lines. The red line indicates the result based on the Monte Carlo algorithm, while the mean rank of each gene of each method is represented in black. Abbreviations: ABA: Abscisic Acid treatment.

Different algorithms yielded different ranking results in this study. RankAggreg was used to determine the final comprehensive ranking list of all genes based on the mSD value of ΔCt, the SD of Bestkeeper, the SV of NormFinder and the M value of geNorm. The results shown as line graphs suggest that the most stable gene combination in different tissues was *AP-2*, *Cpn60β* and *elF-5A* (Figure 6A). In the leaf treated with *Mgl*Flora, *TATA*, *elF-5A* and *RPL17* were the most suitable choices for normalization (Figure 6B), while *TATA*, *HIS* and *ACT2* ranked highest in the treated bud (Figure 6C). Additionally, *EF1α*, *elF-5A* and *TATA* were more stable compared to other genes following ABA treatment (Figure 6D).

GrayNorm is a new algorithm useful for identifying combinations of reference genes. This program provides all combinations of all genes; the number of combinations is reflected as 2^n−1^ (where n is the number of candidate genes). According the lowest number of reference genes required for reliable normalization provided by geNorm, four gene combinations in this study were selected based on the GrayNorm results (Table 6, Table S2). Table 6 shows that a combination of *ACT2*, *HIS* and *TATA* should be used in different tissues. A combination *ACT2*, *EFα* and *HIS* can be used in leaves treated by flowering induction hormone solution, while a combination of *Cpn60β*, *GAPDH* and *HIS* should be used in treated buds. *EF1α*, *HIS* and *TATA* can be used to evaluate ABA-response signaling.

### 2.3. Validation of Selected Candidate Reference Genes

**Figure 7:**
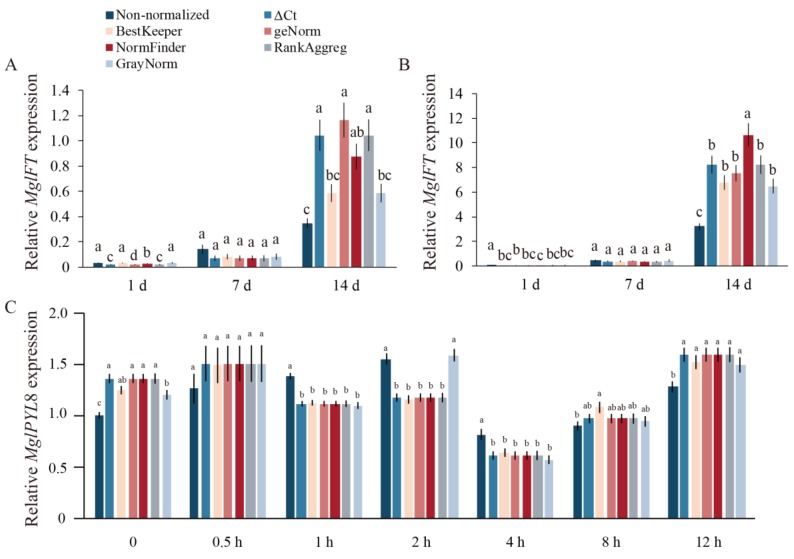
Relative expression levels of two target genes under *Mgl*Flora treatment and ABA treatment. *MgFT* expression levels in leaf of *Mgl*Flora treatment (**A**) and bud of *Mgl*Flora (**B**), *MgPYL8* expression levels under different ABA treatment (**C**), based on the recommendations of six different algorithms. Error bars indicate standard errors of three replicates (bars ± SE).

The relative expression data under *Mgl*Flora treatment are shown in Figure 7. The expression of *MgFT* was not increased sharply until 1 and 7 days after treatment but was significantly changed by 14 days of treatment (Figure 7A). The *MgFT* expression pattern in treated buds was similar to that in the leaf, with expression after 1 day of treatment showing slight upregulation. In the 7 day bud sample set, expression was the same as that on day 1. However, the expression of *MgFT* in the bud was sharply increased after 14 days of treatment and was significantly higher than that under the same conditions used in the treated leaf (Figure 7B). *MgPYL8* expression is shown in Figure 7C. When the expression levels of these two genes were normalized to the different reference gene combinations recommended by the different algorithms, there was a large change in the maximum value of the gene expression and fold-change. Comparing normalized data to non-normalized data, a smaller difference leads to more accurate results. The expression levels of the two genes normalized using the combination recommended by six algorithms showed a small fold-change and the expression level was similar to that of non-normalized genes. 

## 3. Discussion

The selection of stably expressed genes in different tissues and treatments is a key step in qRT-PCR analysis. However, in gymnosperms, the selection of reference genes is limited to *Cycas elongate* [37], *Abies alba* [38] and marine pine [39]. *Metasequoia* originated in the Mesozoic Cretaceous and was declared extinct on Earth until 1941, when Chinese botanists discovered traces of this ancient and rare species in Hubei province, which had been preserved during the Quaternary Ice Age. *Metasequoia* is referred to as a “living fossil” and is a unique tree species in China that has not evolved and its growth process has not diverged. Few studies have examined reference genes in *Metasequoia*. Therefore, we evaluated the stability of the expression of candidate reference genes under specific conditions and then analyzed the usefulness of these genes as internal controls to normalize the expression of other genes. 

In our study, 14 candidate reference genes were successfully identified and their expression characteristics were further analyzed using six statistical algorithms: ΔCt, Bestkeeper, NormFinder, geNorm, RankAggreg and GrayNorm. As shown in Figure 1 and Figure 2, each candidate reference gene showed different Ct values in all samples. The stability of each gene in different samples was clearly observed. *ACT2* and *TATA* exhibited the most stable expression pattern. Our data demonstrate that *TATA* had the highest rank in six tissues and following different hormone treatments. The TATA-box binding protein interacts with the TATA-box, which was the first promoter identified in eukaryotes. The TATA-box is relatively fixed in eukaryotic promoters and plays an important role in regulating the initiation of gene transcription [40]. Notably, the TATA-box binding protein is preferable to other commonly used genes for qRT-PCR analysis in various species, such as, mouse [41], human [42] and *Aphis glycines* [43]. Recently, Izabela also recommended using this gene for normalization in molecular studies of primary and secondary dormancy in *Avena fatua* L [44]. An important feature of an ideal reference gene is that its expression should be constant regardless of the environmental and experimental conditions. However, recent studies showed that widely used reference genes are only stably differentially expressed under certain conditions. *TUB* was predicted to be an unstable reference gene in this study. *TUB* is a member of the tubulin gene family, which are commonly used as reference genes [45] but in *Metasequoia*, its expression stability was low in different tissues and hormone treatment. *TUB* showed lower stable expression than several other reference genes under abiotic stresses in Seashore Paspalum [46]. The results show that not all common reference genes are suitable as reference genes without selection and validation and the same reference genes may have different expression characteristics in different tissue samples and experimental conditions.

Notably, several *Metasequoia* reference genes exhibited different expression patterns compared to in other species treated with ABA. It was previously reported that *UBQ* is the most stable reference gene in NaCl-treated and ABA-treated *Platycladus orientalis* [45]; however, *UBQ* exhibited unstable expression following ABA treatment in this study. Several other widely used reference genes, including *EF1α*, *elF-5A* and *TATA*, were relatively better reference genes for ABA. In previous studies, *TATA* was used as a stable reference gene under heavy metal stress [47] but few reports have examined its expression under abiotic stress. *EF1α* is a commonly used reference gene and was shown to be one of the best reference genes for ABA in *Populus euphraticarice* [8]. Additionally, *EF1α* was selected as best choice for *Petunia hybrida* [48], soybean [49] and *B. brizantha* [50]. This indicates that the reference gene verified in *Metasequoia* is identical to previously reported reference genes. We focused on the influence of hormones on gene expression patterns. Evaluation of hormone treatment revealed the large impact of ABA on gene expression (Table 5, Figure 5D,H). ABA treatment is a type of abiotic stress that leads to activation of numerous stress-related genes and the synthesis of multiple functional proteins in plants. The expression patterns of these reference genes are likely to be greatly affected during this process. *TATA* and *elF-5A* are expressed stably during the flowering stage and are induced by hormone treatment in the leaves and in ABA-treated leaves, suggesting that the expression of these two genes in leaves is not affected by hormones, although different types of hormones have little effects on their expression in the leaves. In future studies, *TATA* and *elF-5A* can be used as valuable reference genes in qRT-PCR assays of hormone treatment in *Metasequoia*. Additionally, *HIS* and *ACT2*, which are also stably expressed in treated buds, did not show stable expression in the treated leaves. These results demonstrate that different tissues under the same treatment conditions exhibit variable gene expression patterns. Our research highlights the importance of choosing suitable reference genes for different tissues.

The expression levels of the target genes were significantly changed when different reference genes were used for normalization, leading to unreliable experimental results. To identify stably expressed reference genes and compare the advantages of these different algorithms, we analyzed and compared the relative expression levels of two functional genes following different hormone treatments. The results revealed numerous differences in the calculated expression patterns of these two target genes when different combinations of reference genes were used. Our results in Figure 7 support that a combination of reference genes is preferred rather than a single internal reference gene. The results of this study provide important information on ABA-response signaling in gymnosperms. Gymnosperms have large genomes but their genome-wide information is limited and the current results provide appropriate resources for qRT-PCR analysis in other species related to *Metasequoia*.

## 4. Materials and Methods 

### 4.1. Hormone Treatment and Sample Collection 

*Metasequoia* trees located on the campus of Beijing Forestry University (latitude 40.0 N, longitude 116.2 E, 57 m above sea level) were growing in the natural environment. Different tissues were collected at 17:00, including leaves, stems, roots, buds, male cores and female cores. After collections, all samples were immediately frozen in liquid nitrogen and stored at −80 °C until RNA extraction.

*Metasequoia* flowering induction hormone solution contained indole butyric acid, Zeatin ribonucleoside (Sigma-Aldrich, St. Louis, MO, USA) and 0.5% Tween-20 [51]. Current-year leaves and buds were treated with hormone solution, which we named as *Mgl*Flora. The treatment was conducted on three *Metasequoia* trees and each represented a biological replicate. The treated shoots were evenly sprayed with *Mgl*Flora until liquid drops began to drip down from the leaves at 17:00 on 11 July 2017. In this treatment, mature leaves and buds from the same position were collected at 1, 7 and 14 days after treatment. For ABA treatment, the leaves were sprayed with 200 μM ABA (Sigma-Aldrich, St. Louis, MO, USA) solution at 17:00 on 11 July 2017. Leaves from the same position were collected at 0, 0.5, 1, 2, 4, 8 and 12 h after treatment. After collection, all samples were immediately frozen in liquid nitrogen and stored at −80 °C until RNA extraction. The average temperature in Beijing Forestry University from July 21 to 25, 2017 is 28.28 °C; the average rainfall during this period is about 170 mm. 

### 4.2. Selection of Candidate Reference Genes and Primer Design

In a previous study, the first large-scale dataset of expressed sequence tags (ESTs) of *Metasequoia* from vegetative and reproductive tissues was reported using 454 pyrosequencing technology [52]. Based on the ESTs, 14 putative reference genes were selected as candidates for qRT-PCR normalization and two target genes were selected to investigate the effect of the choice of reference genes on normalization.

The genes selected were *ACT2* (ACTIN 2), *AP-2* (AP-2 complex subunit mu-like), *Cpn60β* (60-kDa chaperonin β-subunit), *EF1α* (elongation factor-1 α), *elF-5A* (eukaryotic initiation factor 5A), *GAPDH* (eukaryotic initiation factor 5A), *GII*α (glucosidase II α-subunit), *HIS* (histone superfamily protein H3), *RA* (rubisco activase), *RP* (ribosomal L27e protein family), *RPL17* (ribosomal protein L17), *TATA* (TATA binding protein 2), *TUB* (tubulin β chain), *UBQ* (ubiquitin family 6), *FT* (FLOWERING LOCUS T.) and *PYL* (pyrabactin resistance-like8). All primers of these candidate reference genes were designed by Integrated DNA Technologies (http://sg.idtdna.com/calc/analyzer; Coralville, IA, USA). These primers produced amplicons of 150–250 bp, primer lengths were 18–32 bp, optimal Tm (annealing temperature) was 60 ± 2°C and GC% was between 40 and 60. Detailed information for these primers is shown in Table 1 including the primer sequences and annealing temperature of the 14 candidate reference genes. Primer specificity was confirmed by melting-curve analysis after RT-PCR and amplicon sizes were confirmed by 2.0% agarose gel electrophoresis.

### 4.3. Total RNA Isolation and cDNA Synthesis

Frozen samples were ground into a fine powder under liquid nitrogen with a pestle and mortar. Total RNA was isolated from all samples using cetyltrimethyl ammonium bromide (CTAB) buffer according to the instructions [53]. Dithiothreitol (DTT, Thermo Fisher Scientific, Waltham, MA, USA) is a strong denaturant that impacts RNase A (Thermo Fisher Scientific, Waltham, MA, USA) enzyme activity and free thiol, thereby reducing the RNase A enzyme content. DTT was added to the CTAB buffer to improve the extraction efficiency. After chloroform/isoamyl alcohol extraction, RNA was precipitated with 10 M LiCl_2_, followed by extraction with SSTE buffer (1 M NaCl, 0.5% SDS, 10 mM Tris–HCl, 1 mM EDTA) and precipitated with ethanol again. Finally, 75% alcohol were used to clean the RNA precipitate. After the remaining alcohol was evaporated, all RNA samples were dissolved in RNA-free water for subsequent cDNA synthesis experiments. RNA quantity and purity were confirmed by measuring the optical density at OD260/OD280 and OD260/OD230 absorption ratio using a NanoDrop 2000 spectrophotometer (Thermo Fisher Scientific, Waltham, MA, USA). The integrity of the total RNA was checked on 0.8% agarose gels. After adjusting each of the RNA samples to the same concentration, 1000 ng RNA samples were prepared for the synthesis of cDNA strands. cDNA synthesis was performed using PrimerScript RT Enzyme (TaKaRa, Shiga, Japan) following the manufacturer’s instructions.

### 4.4. qRT-PCR Conditions and Amplification Efficiency

qRT-PCR was performed on a Roche 480 light Cycler instrument (Roche, Basel, Switzerland) using TB Green^™^ *Premix Ex Taq*^™^ II (Tli RNaseH Plus) (TaKaRa, Shiga, Japan). The reaction mixture was a total of 20 µL and contained 1 μL cDNA, 10 μL 2× TB Green^™^ *Premix Ex Taq*^™^ II (Tli RNaseH Plus), 0.6 μL 10 μM forward primer, 0.6 μL 10 μM reverse primer and 7.8 μL ddH_2_O. The amplification conditions were as follows: 95 °C for 10 s; 45 cycles at 95 °C for 5 s; 60 °C for 30 s; and 72 °C for 30 s. Each assay included three technical replicates. A standard curve was used to calculate the PCR efficiency (E) for each gene by qRT-PCR using a five-fold dilution series of the mixed cDNA template. The PCR efficiency was calculated by the equation: E = 10^−1/slope^, where slope indicates the slope of a standard curve was given by LightCycler software (Roche, Basel, Switzerland).

### 4.5. Statistical Analysis

The expression stabilities of the 14 reference genes in different tissues and hormone treatments were evaluated using six algorithms: ΔCt [25], Bestkeeper [54], NormFinder [23], geNorm [22], RankAggreg [27] and GrayNorm [26].

The principle of the ΔCt algorithm is to compare the relative expression of the ‘gene pair’ in each sample to identify the reference gene that can be stably expressed. The difference in Ct (ΔCt) between the two genes is first calculated. If the ΔCt of the two genes in different cDNA samples remains the same, these two genes were considered as stably expressed. The standard deviation (sd) reflects the change in the ΔCt of the two genes in all samples. Next, the ΔCt value between one gene and the other 13 genes was calculated and the 13 SD values were averaged to obtain the mean standard deviation (mSD).

BestKeeper is a tool that determines the stable level of reference genes based on pairwise correlations calculated from the percentage standard deviation (SD) and covariance (CV) among the raw Ct value data. Candidate reference genes showing the lowest CV values were considered as the most stable genes. Another function of the BestKeeper program is that it applies the decision coefficient (r) as an index of the program’s stability characterization. r was calculated to determine a credible normalization factor (NF), rather than evaluating the excellence of each reference gene’s expression stability separately. In this study, we used r to rank the stability values of the 14 candidate genes.

NormFinder values the gene stability based on the expression variations of these candidate reference genes, with a lower stability value indicating more stable reference genes. The algorithm calculates inter-group and intra-group changes in response to the NF, with the algorithm requiring at least three candidate genes and more than two samples per group.

geNorm analyzes the expression stability of a candidate reference gene according the stability value (M, M-value), with smaller M values indicating better stability. The standard of selected reference genes was based on the M value, with values of less than 1.5 considered to indicate stable expression of an ideal reference gene. Additionally, geNorm also valued the pairwise variation values (V), indicating the lowest number of reference genes required for reliable normalization. 

GrayNorm is an algorithm that can identify a combination of reference genes with minimal deviation from non-normalized data. The principle of GrayNorm is to calculate the NF for each combination of treatment group and each possible reference gene. The closer the mean value of 1/NF for each treatment group is to 1.0, the smaller the biological variation and the more accurate the expression level of gene of interest can be calculated.

The R program v3.0.1 can load the RankAggreg v0.6.4 package. RankAggreg meets the needs of complex rank aggregation easily and expediently, integrating the stability measurements obtained from the four methods and then establishes a comprehensive ranking of reference genes. RankAggreg contains various algorithms, such as Cross-Entropy Monte Carlo algorithm, Genetic algorithm and a brute force algorithm. The detailed algorithm process can be referenced at https://cran.r-project.org/web/packages/RankAggreg/RankAggreg.pdf. According to the ranking orders of these four methods, the Cross-Entropy Monte Carlo algorithm (CE) was used to demonstrate hierarchical aggregation. The result was intuitively output in the form of a line graph. 

## 5. Conclusions

Stable reference genes are used to determine the relative gene expression levels and show stable expression under different treatment conditions for qRT-PCR. Genes utilized as qRT-PCR candidate reference genes have not been reported for *Metasequoia*. This study was conducted to select reference genes after evaluating their expression stability in various tissues and hormone conditions. The results of this study emphasize the importance of validating reference genes for qRT-PCR analysis in *Metasequoia*.

## Figures and Tables

**Table 1:** Primer sequences and PCR amplification characteristics of the 14 selected reference genes and two target genes.

Gene Abbreviation	Primer Sequence of Forward (5′–3′)	Primer Sequence of Reward (5′–3′)	Amplicon Length (bp)	Tm(°C)	E	Arabidopsis Ortholog No.
*ACT2*	GACGCTTATGTTGGTGATGAGGC	GAGTCATCTTCTCTCTGTTTGCCTTAGG	211	58.2 °C/58.4 ºC	1.9807	AT5G09810.1
*AP-2*	GGAAGTGTTCTCCGGTGCGATG	CAGTTCAAATTCTCCATCAGGTGGGAC	252	60.3 °C/59.3 °C	2.1419	AT5G46630.2
*Cpn60β*	GTGATCGCGCCAGAATGGCATC	CCGACCATAACTTTGGCAGCAGG	196	61.1 °C/60.5 °C	1.9454	AT5G56500.1
*EF1α*	GCTTGGGTGCTTGACAAGCTCAAG	CAGAGCATGTTCTCGGGTCTGTCC	251	60.8 °C/61.2 °C	1.9384	AT5G60390.1
*elF-5A*	GTCGGATGAGGAGCACCATTTTGATCAC	CAGTTGTGGGATGAAGGAACTATATCCTCG	245	61.1 °C/59.7 °C	2.0517	AT1G13950.1
*GAPDH*	GATGATGTCGAGCTCGTTGCAGTGAAC	GATTCAACCACATACTCTGCACCAACC	224	61.3 °C/59.7 °C	2.0367	AT1G13440.1
*GIIα*	CGGTCCCCAGGCTGTTAGTTTAGATG	CGTCGTCGACTCCTTGGAATGAGAG	229	60.8 °C/60.6 °C	1.9042	AT5G63840.1
*HIS*	CACAGATACCGTCCCGGAACTGTTG	GCTTCTGCAGCTTCCTGGAGAGC	179	61.1 °C/62.2 °C	2.0234	AT4G40030.2
*RA*	GATGAGTGCGGGAGAGCTTGAAAGTG	GATGAGTGCGGGAGAGCTTGAAAGTG	138	61.4 °C/61.4 °C	1.9410	AT2G39730.1
*RP*	GGTCACTGCCTCGTCGCAG	GCCTTCAGATCCACATCCAATGTGTG	164	61 °C/59.9 °C	1.8518	AT3G22230.1
*RPL17*	GAGGCAGCCAATGGCACTCATC	CAACCTGGTTGAAGGTCTTCCCATTG	167	60.9 °C/59.9 °C	1.9946	AT1G04270.1
*TATA*	GGAAGGGAGTCAGCCTGTCGATCTG	GCACCTGTGCAGACCATCTTTCCTGAT	229	62.5 °C/62.6 °C	1.9278	AT1G55520.1
*TUB*	GTTTGAGGTTCCCTGGTCAGCTC	CTGCCATGTCTTGGATCAGCAGC	211	59.8 °C/60.6 °C	2.0385	AT5G23860
*UBQ*	CGGCCGTACTCTTGCCGAC	GGCCTTGACGTTGTCGATGGTG	157	61 °C/60.7 °C	1.9534	AT5G20620.1
*FT*	GTCACGAGATCCACTAACGACGGG	GCTTGAGCTCGCAGCCATTTTTG	125	60.8 °C/59.7 °C	1.8475	AT1G65480.1
*PYL8*	CAGCGACAGCTAGCGAAGAGAG	CCATTGTACCTGGCCTCCCATC	154	59.5 °C/59.8 °C	1.9692	AT5G53160.2

Abbreviations: E: PCR efficiency.

**Table 2:** Candidate reference gene ranking based on ΔCt method.

Rank	Total	mSD	Tissue	mSD	LEAF of *Mgl*Flora	mSD	Bud of *Mgl*Flora	mSD	ABA	mSD
1	*EF1α*	0.522	*Cpn60β*	1.41	*TATA*	0.75	*TATA*	0.74	*elF-5A*	1.18
2	*Cpn60β*	0.522	*EF1α*	1.43	*elF-5A*	0.77	*ACT2*	0.78	*EF1α*	1.19
3	*TATA*	0.698	*elF-5A*	1.43	*RPL17*	0.81	*EF1α*	0.83	*TATA*	1.19
4	*HIS*	0.761	*AP-2*	1.44	*EF1α*	0.82	*HIS*	0.84	*ACT2*	1.21
5	*RPL17*	0.806	*ACT2*	1.53	*GAPDH*	0.84	*RPL17*	0.86	*Cpn60β*	1.26
6	*ACT2*	0.884	*GIIα*	1.58	*UBQ*	0.87	*RA*	0.86	*RPL17*	1.27
7	*RP*	0.949	*RPL17*	1.6	*RA*	0.92	*GIIα*	0.89	*RP*	1.29
8	*elF-5A*	1.006	*RP*	1.66	*GIIα*	0.93	*TUB*	0.99	*AP-2*	1.32
9	*AP-2*	1.049	*HIS*	1.69	*TUB*	0.94	*UBQ*	1.02	*HIS*	1.38
10	*GAPDH*	1.118	*TATA*	1.75	*HIS*	1.09	*Cpn60β*	1.03	*GAPDH*	1.76
11	*GIIα*	1.267	*GAPDH*	1.92	*Cpn60β*	1.09	*GAPDH*	1.12	*RA*	2.4
12	*UBQ*	1.596	*UBQ*	2.37	*RP*	1.16	*elF-5A*	1.12	*GIIα*	2.8
13	*RA*	1.869	*RA*	3.93	*AP-2*	1.68	*RP*	1.18	*UBQ*	3.79
14	*TUB*	2.189	*TUB*	4.01	*ACT2*	2.23	*AP-2*	1.8	*TUB*	4.32

Abbreviations: mSD: mean standard deviation; ABA: Abscisic Acid treatment.

**Table 3:** BestKeeper-based expression stability evaluation of 14 candidate reference genes.

Rank	Total	r	Tissue	r	Leaf of *Mgl*Flora	r	Bud of *Mgl*Flora	r	ABA	r
1	*HIS*	0.944	*AP-2*	0.969	*HIS*	1.000	*Cpn60β*	1.000	*TATA*	0.989
2	*TATA*	0.913	*HIS*	0.960	*ACT2*	0.996	*GIIα*	0.999	*Cpn60β*	0.964
3	*ACT2*	0.889	*TATA*	0.927	*TUB*	0.994	*HIS*	0.998	*elF-5A*	0.936
4	*Cpn60β*	0.834	*EF1α*	0.927	*TATA*	0.99	*EF1α*	0.998	*EF1α*	0.923
5	*RPL17*	0.818	*ACT2*	0.903	*EF1α*	0.989	*TATA*	0.998	*HIS*	0.909
6	*EF1α*	0.800	*Cpn60β*	0.808	*Cpn60β*	0.975	*ACT2*	0.997	*ACT2*	0.845
7	*GAPDH*	0.750	*GAPDH*	0.788	*UBQ*	0.974	*GAPDH*	0.993	*RPL17*	0.794
8	*AP-2*	0.648	*GIIα*	0.777	*GIIα*	0.938	*RA*	0.985	*AP-2*	0.687
9	*GIIα*	0.613	*RP*	0.776	*elF-5A*	0.931	*elF-5A*	0.984	*RP*	0.658
10	*RP*	0.588	*RPL17*	0.763	*GAPDH*	0.902	*RPL17*	0.977	*GAPDH*	0.491
11	*elF-5A*	0.503	*elF-5A*	0.442	*RA*	0.897	*TUB*	0.960	*RA*	0.452
12	*UBQ*	0.426	*TUB*	0.363	*RPL17*	0.874	*UBQ*	0.956	*GIIα*	0.153
13	*TUB*	0.276	*UBQ*	0.158	*RP*	0.79	*RP*	0.908	*UBQ*	0.001
14	*RA*	0.138	*RA*	0.130	*AP-2*	0.001	*AP-2*	0.856	*TUB*	0.001

Abbreviations: ABA: Abscisic Acid treatment; r: correlation coefficient.

**Table 4:** Expression stability values of the 14 candidate reference genes calculated using geNorm.

Rank	Total	M	Tissue	M	Leaf of *Mgl*Flora	M	Bud of *Mgl*Flora	M	ABA	M
1	*EF1α*	0.522	*AP-2*	0.487	*RPL17*	0.105	*EF1α*	0.19	*EF1α*	0.139
2	*Cpn60β*	0.522	*Cpn60β*	0.487	*elF-5A*	0.105	*HIS*	0.19	*TATA*	0.139
3	*TATA*	0.698	*elF-5A*	0.542	*GAPDH*	0.147	*ACT2*	0.243	*elF-5A*	0.155
4	*HIS*	0.761	*EF1α*	0.622	*TATA*	0.284	*TATA*	0.294	*ACT2*	0.195
5	*RPL17*	0.806	*ACT2*	0.692	*TUB*	0.377	*GIIα*	0.34	*Cpn60β*	0.227
6	*ACT2*	0.884	*RP*	0.772	*UBQ*	0.406	*Cpn60β*	0.371	*RPL17*	0.296
7	*RP*	0.949	*GIIα*	0.823	*EF1α*	0.472	*RA*	0.449	*RP*	0.342
8	*elF-5A*	1.006	*RPL17*	0.88	*RA*	0.55	*GAPDH*	0.535	*AP-2*	0.376
9	*AP-2*	1.049	*HIS*	0.964	*GIIα*	0.592	*RPL17*	0.627	*HIS*	0.428
10	*GAPDH*	1.118	*TATA*	1.018	*Cpn60β*	0.648	*UBQ*	0.694	*GAPDH*	0.564
11	*GIIα*	1.267	*GAPDH*	1.079	*HIS*	0.689	*TUB*	0.756	*RA*	0.816
12	*UBQ*	1.596	*UBQ*	1.258	*RP*	0.75	*elF-5A*	0.81	*GIIα*	1.113
13	*RA*	1.869	*RA*	1.645	*AP-2*	0.87	*RP*	0.872	*UBQ*	1.48
14	*TUB*	2.189	*TUB*	1.983	*ACT2*	1.065	*AP-2*	1.005	*TUB*	1.885

Abbreviations: ABA: Abscisic Acid treatment; M: M-values.

**Table 5:** NormFinder-based expression stability evaluation of 14 candidate reference genes.

Rank	Total	SV	Tissue	SV	Leaf of *Mgl*Flora	SV	Bud of *Mgl*Flora	SV	ABA	SV
1	*RPL17*	0.428	*AP-2*	0.197	*TATA*	0.152	*TATA*	0.233	*TATA*	0.070
2	*EF1α*	0.448	*Cpn60β*	0.282	*elF-5A*	0.210	*RPL17*	0.366	*EF1α*	0.070
3	*Cpn60β*	0.478	*elF-5A*	0.352	*EF1α*	0.249	*ACT2*	0.389	*elF-5A*	0.080
4	*RP*	0.582	*GIIα*	0.454	*RPL17*	0.355	*RA*	0.435	*ACT2*	0.085
5	*TATA*	0.652	*EF1α*	0.582	*UBQ*	0.365	*EF1α*	0.581	*Cpn60β*	0.120
6	*HIS*	0.670	*ACT2*	0.683	*GAPDH*	0.415	*HIS*	0.613	*RPL17*	0.177
7	*ACT2*	0.782	*RP*	0.810	*TUB*	0.527	*GIIα*	0.663	*RP*	0.177
8	*AP-2*	0.921	*RPL17*	0.862	*RA*	0.561	*TUB*	0.673	*AP-2*	0.184
9	*elF-5A*	0.948	*HIS*	1.038	*GIIα*	0.590	*UBQ*	0.697	*HIS*	0.398
10	*GAPDH*	1.186	*TATA*	1.206	*HIS*	0.773	*elF-5A*	0.841	*GAPDH*	1.031
11	*GIIα*	1.770	*GAPDH*	1.472	*Cpn60β*	0.824	*Cpn60β*	0.897	*RA*	2.072
12	*UBQ*	3.104	*UBQ*	1.793	*RP*	0.970	*GAPDH*	0.943	*GIIα*	2.474
13	*RA*	3.335	*RA*	3.730	*AP-2*	1.621	*RP*	0.967	*UBQ*	3.726
14	*TUB*	3.886	*TUB*	3.839	*ACT2*	2.180	*AP-2*	1.748	*TUB*	4.255

Abbreviations: ABA: Abscisic Acid treatment; SV: stability values.

**Table 6:** Best combinations of reference genes based on ΔCt, BestKeeper, Normfinder, geNorm, RankAggreg or GrayNorm in different tissues and different hormone treatments.

Algorithms	Tissue	Leaf of *Mgl*Flora Treatment	Bud of *Mgl*Flora Treatment	ABA
Delta CT	*Cpn60β* + *EF1α* + *elF-5A*	*TATA + elF-5A + RPL17*	*TATA + ACT2 +EF1α*	*elF-5A + TATA + EF1α*
BestKeeper	*HIS* + *EF1α* + *TATA*	*HIS + ACT2 + EF1α*	*Cpn60β + HIS + EF1α*	*TATA + EF1α + Cpn60β*
NormFinder	*AP-2* + *Cpn60β* + *elF-5A*	*TATA + elF-5A + EF1α*	*TATA + RPL17 + ACT2*	*TATA + EF1α + elF-5A*
geNorm	*AP-2 + Cpn60β + elF-5A*	*RPL17 + elF-5 + GAPDH*	*EF1α + HIS + ACT2*	*EF1α + TATA + elF-5A*
RankAggreg	*AP-2 + Cpn60β + elF-5A*	*TATA + elF-5A + RPL17*	*TATA + HIS + ACT2*	*EF1α + elF-5A + TATA*
GrayNorm	*ACT2 + HIS + TATA*	*ACT2 + EFα + HIS*	*Cpn60β + GAPDH + HIS*	*EF1α + HIS + TATA*

## Supplementary Materials

Supplementary materials can be found at http://www.mdpi.com/1422-0067/20/1/34/s1.
