# Supplementary figures and images for: Comparison of Reliable Reference Genes Following Different Hormone Treatments by Various Algorithms for qRT-PCR Analysis of Metasequoia
Source: Int J Mol Sci. 2018 Dec 21;20(1):34. doi: 10.3390/ijms20010034 (PMC6337471; doi:10.3390/ijms20010034)

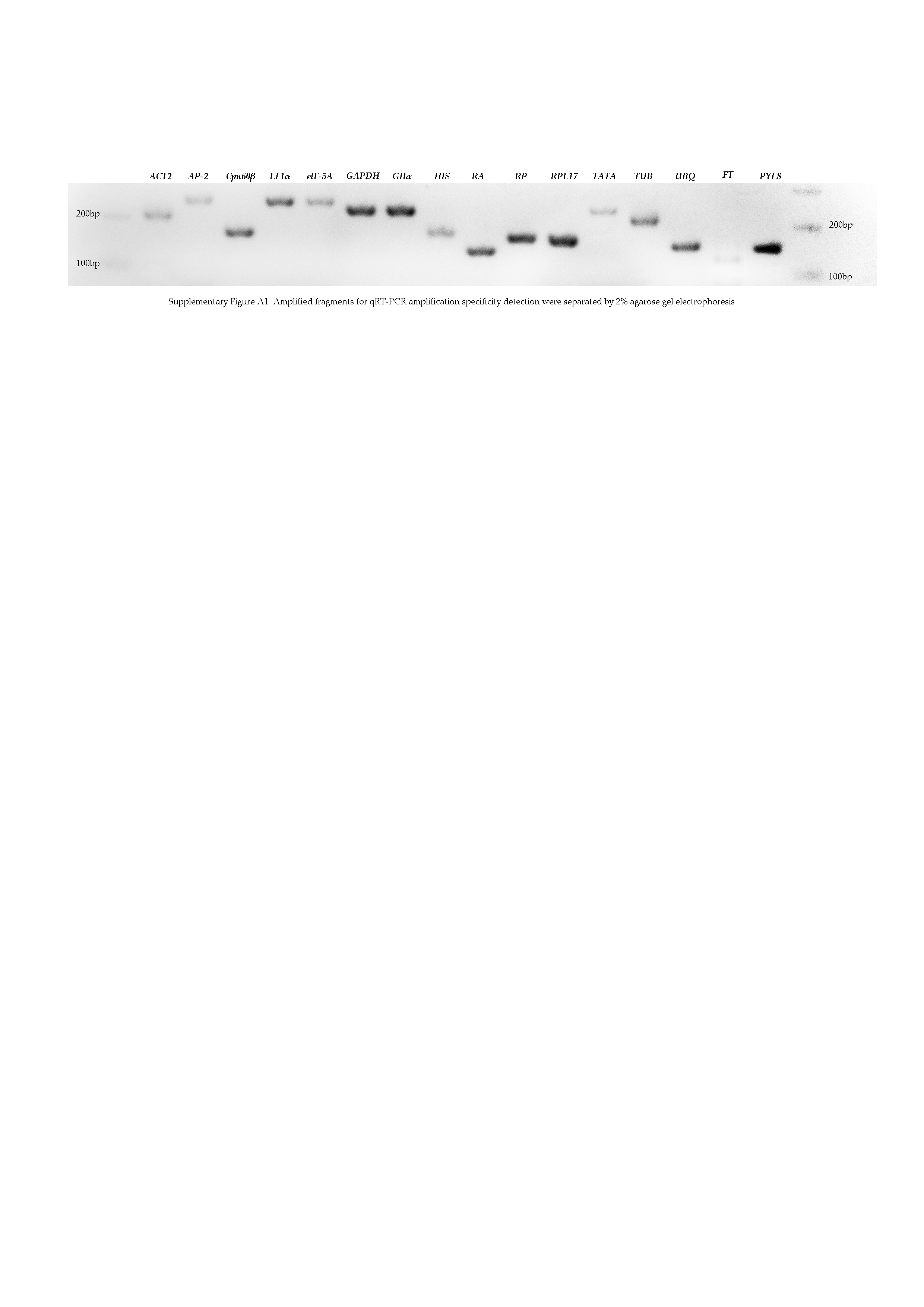

Supplement: Supplementary file 1 [file ijms-20-00034-s001.zip › Supplementary Figure A1.tif]

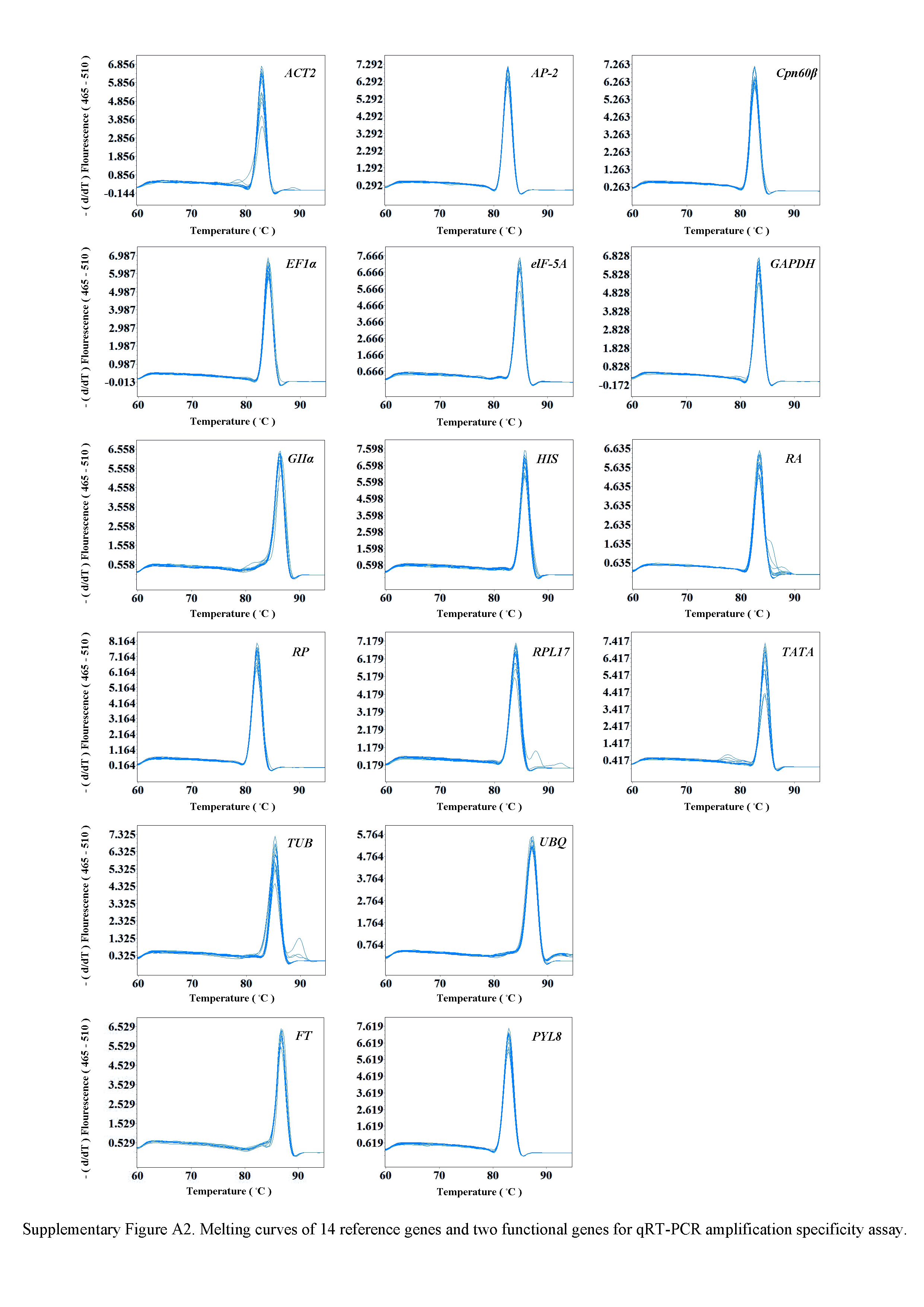

Supplement: Supplementary file 1 [file ijms-20-00034-s001.zip › Supplementary Figure A2.tif]
